# Formulation film based on zinc acetate/cellulose with anti-inflammatory, angiogenesis, and remodeling abilities for enhancing burn healing

**DOI:** 10.1038/s41598-025-27021-w

**Published:** 2026-01-02

**Authors:** Rania S. Salah, Ghada H. Elsayed, Marwa El-Hussieny, Mohamed R. Mousa, Sawsan Dacrory

**Affiliations:** 1 Hormones Department, Medical Research and Clinical Studies Institute, National Research Centre, Dokki, Giza, 12622 Egypt; 2 Stem Cells Lab, Centre of Excellence for Advanced Sciences, National Research Centre, Dokki, Giza, 12622 Egypt; 3 Organometallic and Organometalloid Chemistry Department, National Research Centre, Dokki, Giza, 12622 Egypt; 4 Pathology Department, Faculty of Veterinary Medicine, Cairo University, Giza, 12211 Egypt; 5 Cellulose and Paper Department, National Research Centre, Dokki, Giza, 12622 Egypt

**Keywords:** Skin burn, Cellulose formulation film, Inflammation, Angiogenesis, Remodeling, Biochemistry, Biotechnology, Diseases, Drug discovery, Medical research

## Abstract

Burns are most challenging to treat as they result in skin impairment or even death. This study elucidated the therapeutic benefits of Zinc/cellulose films on thermal burn injuries in rats at two distinct time points [eight and sixteen days after the injury]. A novel formulation film based on Zinc acetate and cellulose for enhancing burn healing has been prepared. Hydroxyl ethyl cellulose (HEC) and Polyvinyl alcohol have mixed to form homogenous solution then zinc acetate with different ratios has been added. The prepared film has been investigated by different analysis FTIR, XRD and SEM then applied on burn rat model using Silvazine cream as a reference drug. Our findings illustrated that treatments under investigation after 8 and 16 days enhanced wound contraction rate and improved pathological alteration triggered by burn induction. C-Zn1.5 film declined IL-1β [8 day 30.03 ± 0.24, *p<0.001*; 16 day 18.43 ± 0.31, *p<0.001*] and TNF-α [8 day 68.14 ± 0.54, *p<0.001*; 16 day 39.35 ± 0.55, *p<0.001*] levels, and increased collagen1 [8 day 3.92 ± .08, *p<0.001*; 16 day 6.06 ± .15, *p<0.001*] and Bcl2 [8 day 277.7 ± 1.26, *p<0.001*; 16 day 396.27 ± 2.07, *p<0.001*] concentrations at 8 and 16 days in burn tissues. Additionally, the C-Zn1.5 film downregulated the expression levels of VEGF (~90%), TGF-β (~91%), MMP2 (~85%), and TIMP2 (~88%) genes at day 8; however, they upregulated gene levels of VEGF (~340%), TGF-β (~313%), MMP2 (~413%), and TIMP2 (~259%) at day 16 in skin burnt tissues. Collectively, Zinc/cellulose 1.5 films was superior in promoting burn wound healing via mechanisms possibly associated with its anti-inflammatory, anti-apoptotic, angiogenesis, and remodeling abilities.

## Introduction

Burn incidence is intimately related to emergencies since severe burns can be fatal^1^**.** It could be induced by heat, electricity, radiation, and exposure to certain chemicals^2^**.** According to wound depth and the size of the injured skin region, that assessed as the percentage of total body surface area, burn injuries are frequently categorized into superficial, partial-thickness, and full-thickness burns^3^**.** Among several topical treatments choices for partial-thickness burns is silver-sulfadiazine cream; however, serious burns, on occasion, need skin grafting surgery^4,5^**.**

Subsequent thermal injury, localized tissue damage and a massive systemic inflammatory response are instigated by significant alterations in the local tissue microenvironment and consequent macrophage phenotype^6^***.*** Despite the wound recuperation process following thermal injury being lengthy; it commonly comprises three interrelated and overlapping stages including inflammation, proliferation, and remodeling. Each of these phases involves the cooperative contribution of various repair cells, inflammatory cells, growth factors, and extracellular matrix components^6,7^***.*** Inflammation is the earliest stage and can last roughly five to seven days. It is epitomized by neutrophils and macrophages recruitment to the trauma site, under the chemotaxis of inflammatory factors^8^. Prolonged inflammation can impede wound healing, which is regulated by several proteins and cytokines, including IL-4, IL-10, IL-13, and TGF-β^9^. Achieving a balance between pro-inflammatory and anti-inflammatory cytokines is crucial for effective burn wound healing. Pro-inflammatory cytokines, such as tumor necrosis factor-alpha (TNF-α), interleukin-1 beta (IL-1β), and interleukin-6 (IL-6), are released in burn wounds, leading to tissue damage and delayed healing^10^.

The proliferative phase, also known as the granulation phase, occurs three to ten days post-injury. It is distinguished by creation of new capillaries and is crucial for proliferation and differentiation of vascular endothelial cells^11^***.*** Furthermore, fibroblasts proliferate during this phase to create new collagen, which significantly stimulate formation of granulation tissue^7,12^ . Finally, the mature phase- also referred to as the remodeling phase- may take up to a year to fully recover after injury^6^. Extracellular matrix [ECM], including collagen fibers, is secreted by fibroblasts. Newly synthesized collagen fiber, which is more elastic and regular, gradually replaces the irregular one^13^**.** Any alterations throughout the healing phases lead to the development of excessive scarring and/or chronic ulcers^14^**.**

Conventional formulations for topical and dermatological administration of drugs have certain limitations like poor adherence to skin, poor permeability and compromised patient compliance. For the treatment of diseases of body tissues and wounds, the drug has to be maintained at the site of treatment for an effective period of time. Topical film forming systems are such developing drug delivery systems meant for topical application to the skin, which adhere to the body, forming a thin transparent film and provide delivery of the active ingredients to the body tissue^15^. Nanotherapeutics involving metals and polymers has enormous potential in treating burn wounds^16^.

Cellulose, the most fundamental bio-based polymer, has been used for centuries. It is a potential scaffold material for repairing skin injuries because of its distinct chemical structure, mechanical strength, ease of processing, and flexibility^17^. Zinc (Zn), a trace element that is essential micronutrient in human bodies, plays a crucial role in the process of wound healing^18^***.*** It is necessary for immune system function, cell proliferation and growth, and reparation of cell membrane^19^. It considered a cofactor for numerous metalloenzymes involved in wound healing and dermal regeneration including integrins, matrix metalloproteinases (MMPs), metallothioneins (MT), and Zn finger transcription factors^20,21^**.** Zinc could persuade hemostasis via modulating platelet aggregation, clotting factors, and interaction with endothelial cells^22^***.*** Besides, it suppresses the growth of numerous bacterial species^23^ and regulates early inflammatory responses^24^. It promotes fibroblast proliferation, accelerating ECM synthesis and secretion, and lowers free radicals generation to safeguard cellular viability^25^***.*** Additionally, it can promote collagen synthesis and participate in re-epithelization^23^***.***

While the prognosis for patients has improved due to current burn damage management procedures, there are still major concerns about increasing morbidity and mortality^26^**.** Therefore, identification of the mechanisms underlying burn healing is critical for the development of improved treatment modalities. In the present study, our goal was to illustrate the possible therapeutic advantages of various topical treatments using cellulose, Zinc-cellulose 1, and Zinc-cellulose 1.5 films for thermal burn trauma at two distinct time points 8- and 16-days post-injury.

## Materials and methods

Hydroxyl ethyl cellulose (HEC) medium viscosity, MS between 1.5 and 2.5 and DS between 0.4 to 2.0, was purchased from Fluka. The DS value represents the average number of hydroxyl groups on the cellulose that have been substituted with hydroxyethyl groups, while the MS value indicates the average number of hydroxyethyl groups(–CH_2_CH_2_OH) per anhydroglucose unit. Polyvinyl alcohol (PVA) and zinc acetate have purchased from Shanghai Aladdin Biochemical Technology Co., Ltd. All chemicals and reagents were analytical grade and used without further purification.

### Chemical studies

#### Preparation of film composite

A homogenous solution of HEC/PVA has been prepared by dissolving 5 g of HEC and PVA individually `in 100 ml water with stirring. Then 10 ml of each solution (HEC and PVA) have mixed with continues stirring at room temperature for 30 min. Different ratio of 2% Zn has been added individually (1 and 1.5 ml). The solution has poured in petri dish and allowed to dry at room temperature.

### Characterizations

FT-IR spectra were recorded in the range of 400–4000 cm^−1^ on (Shimadzu 8400S) FT-IR Spectrophotometer. The surface morphology was analyzed using (SEM) electron microscope FEI IN SPECTS Company, Philips, Poland, environmental scanning without coating with a JEOL JEM-2100 electron microscope at 100 k x magnification and an acceleration voltage of 120 kV. The XRD patterns were investigated on a Diano X-ray diffractometer using CuKα radiation source energized at 45 kV and a Philips X-ray diffractometer (PW 1930 generator, PW 1820 goniometer) with CuK radiation source (λ = 0.15418 nm), at a diffraction angle range of 2θ from 10 to 80° in reflection mode.

### In vivo studies

#### Ethical considerations and animals housing

All animal experiments in this study were designed, conducted, and reported in strict accordance with the core principles and reporting checklist of the ARRIVE guidelines (version 2.0). All experiments were performed as the guidelines for the care and use of laboratory animals. Medical Research Ethical Committee of the National Research Centre in Egypt authorized all procedures involving rats. The experiment identification number is 13010104-1. One hundred and twenty healthy female *Wistar* rats, weighing about 200 g, were supplied from the Animal House Colony of the National Research Centre, Egypt. The rats were given unlimited access to food and water, and the animal room was kept at a temperature and humidity 22–24 °C and 50%–60% respectively. They were also exposed to a 12-h light/12-h dark cycle.

### Thermal burn induction

Burn skin injury model was established as earlier reported by Liu et al.^27^. After a week of acclimation, the fur was removed from each rat’s dorsal surface using depilatory cream a day before burn wound creation to avoid excessive stress on the skin. Following isoflurane anesthesia, the back of each rat sterilized with antiseptic iodophor. Then a solid aluminum rod [10 mm in diameter; 51 g] formerly heated by immersing in boiling water (100 °C) for 10 min was placed on the rats’ backs for 15 s^28^***.*** The burn injuries were dressed with an equal sized layer of the prepared films based on their grouping once daily.

### Topical treatment application

Information regarding the studied experimental groups is represented in Table 1. Six groups of animals were formed as follows: Normal control group (NG) unburnt healthy rats; burn group (BG) burnt rat without medication; blank cellulose group (CG) burnt rats covered with blank cellulose films. While, for each burn lesion in Zn-cellulose1 (C-Zn1G) and Zn-cellulose 1.5 (C-Zn1.5G) groups, a daily film of Zn/cellulose was applied respectively. As a reference medication, Silvazine cream (silver sulphadiazine (1%)) was used to treat burn in Silvazine group (SG).

**Table 1:** Animals grouping.

Groups	Descriptions	Animals number for each treatment time	
		8 Days	16 Days
NG	Normal rats	10	10
BG	Untreated burnt rats	10	10
CG	Burnt rats and treated with blank cellulose film	10	10
C-Zn1G	Burnt rats and treated with Zn/cellulose film (2% Zn (1 ml)	10	10
C-Zn1.5G	Burnt rats and treated with Zn/cellulose film (2% Zn (1.5 ml)	10	10
SG	Burnt rats and treated with silvazine cream	10	10
	Total	120 animals	

### Evaluation of burn wound healing

The digital pictures of the burn lesions were taken on days 0, 4, 8, 12 and 16 post-operatives to visualize the wound healing pattern. Then all pictures were evaluated using size analysis software-Image J to estimate area of burn injuries. The wound healing rates of the six burnt rats were expressed as wound contraction rates (WCR) that represents the percent of reduction in the area of original wound and was calculated as follows:

$${\text{Wound contraction rate }}\left( \% \right) \, = \, \left( {{\text{WA}}0 \, - {\text{ WAn}}} \right)/{\text{WA}}0 \, \times { 1}00,$$

where WA0 is the original wound area and WAn is the wound area on day n. According to burn wound healing, all animals were sacrificed on day 16 after burning.

### Skin samples preparation

On days 8 and 16 following burn creation, the rats were subjected to pentobarbital sodium anesthesia and sacrificed by cervical dislocation. Skin samples from the burn wounds were collected, homogenized (20% w/v) and centrifuged at 3000*g* for 10 min at 4 °C after being mixed with an ice-cold medium containing 50 mM PBS (pH 7.4). The obtained supernatants will subsequently be used for the biochemical analysis. Some samples were kept at − 80 °C for using in gene expression analysis, while the others were fixed in neutral buffered formalin for using in histopathological processing.

### ELISA assays

According to the manufacturer’s instructions, the specified rat ELISA kits were used to estimate the concentrations of IL-1beta, TNF-α, collagen 1, and Bcl2 in skin homogenate of six rats. We purchased all of the ELISA kits from Sunlong Biotech Company in China.

### Quantitative real-time PCR (qRT-PCR) analysis

RNAeasy mini-Kit (Qiagen, Germany) was used to separate RNA from skin tissues. The NanoDrop One microvolume UV spectrophotometer (Thermo Fisher Scientific, USA) was then used to measure the concentration and purity of the total extracted RNA. The Revert Aid First Strand cDNA Synthesis Kit (Thermo Fisher Scientific, USA) was used to convert the RNA from each treatment to first-strand cDNA in accordance with the manufacturer’s instructions. Table 2 lists certain primer sequences. Maxima SYBR Green qPCR Master Mix (2X) (Thermo Fisher Scientific, USA) was used to normalize the expression levels of the VEGF, TGF-β, MMP2, and TIMP2 genes with respect to the β-actin transcript, and the 2^−ΔΔCT^ technique was used to compute the results^28^**.** The following reaction conditions were used: 40 cycles of amplification at 95 °C for 10 min, 95 °C for 15 s, 55 °C for 30 s, and 72 °C for 30 s. Thermocycler DT Lite 4S1 DNA Technology Detecting was utilized to quantify gene expression.

**Table 2:** The qRT-PCR analysis primers.

Gene	Forward primer (5′-3′)	Reverse primer (5′-3′)
β-actin	CACGTGGGCCGCTCTAGGCACCAA	CTCTTTGATGTCACGCACGATTTC
VEGF	AGGCTGCACCCACGACAGAA	CTTTGGTCTGCATTCACATC
TGF-β	CTGAACCAAGGAGACGGAAT	GGTTCATGTCATGGATGGTG
MMP2	TTCTGTCCCGACCAAGGA	GGTGTAGATAGGGGCCATCA
TIMP2	CGTTTTGCAATGCAGACGTA	GATGGGGTTGCCATAGATGT

### Hematoxylin–eosin staining

Samples of burned skin tissue were obtained and preserved for 24 h in 10% neutral buffered formalin. Samples immersed in molten paraplast after being dehydrated in escalating grades of ethanol. The blocks were sectioned into pieces using a microtome with 5 µm thick and then stained with hematoxylin and eosin. Slices of tissues were examined at the microscopic level using a microscope^29^.

### Statistical analysis

In this study, Mean ± SEM of the mean was used to express all results. The Statistical Package for the Social Sciences (SPSS) software (version 25) was utilized to analyze biochemical data using one-way analysis of variance (ANOVA), and the least significant difference (LSD) was performed to compare group significance. However, one-way ANOVA and the Tukey Kramer multiple comparison test were used to identify statistically significant differences between groups in the molecular data. GraphPad Prism version 8 was uses for data analysis. For every test, a significant difference was defined as p < 0.05.

## Results and discussion

### Preparation of film composite

**Fig. 1:**
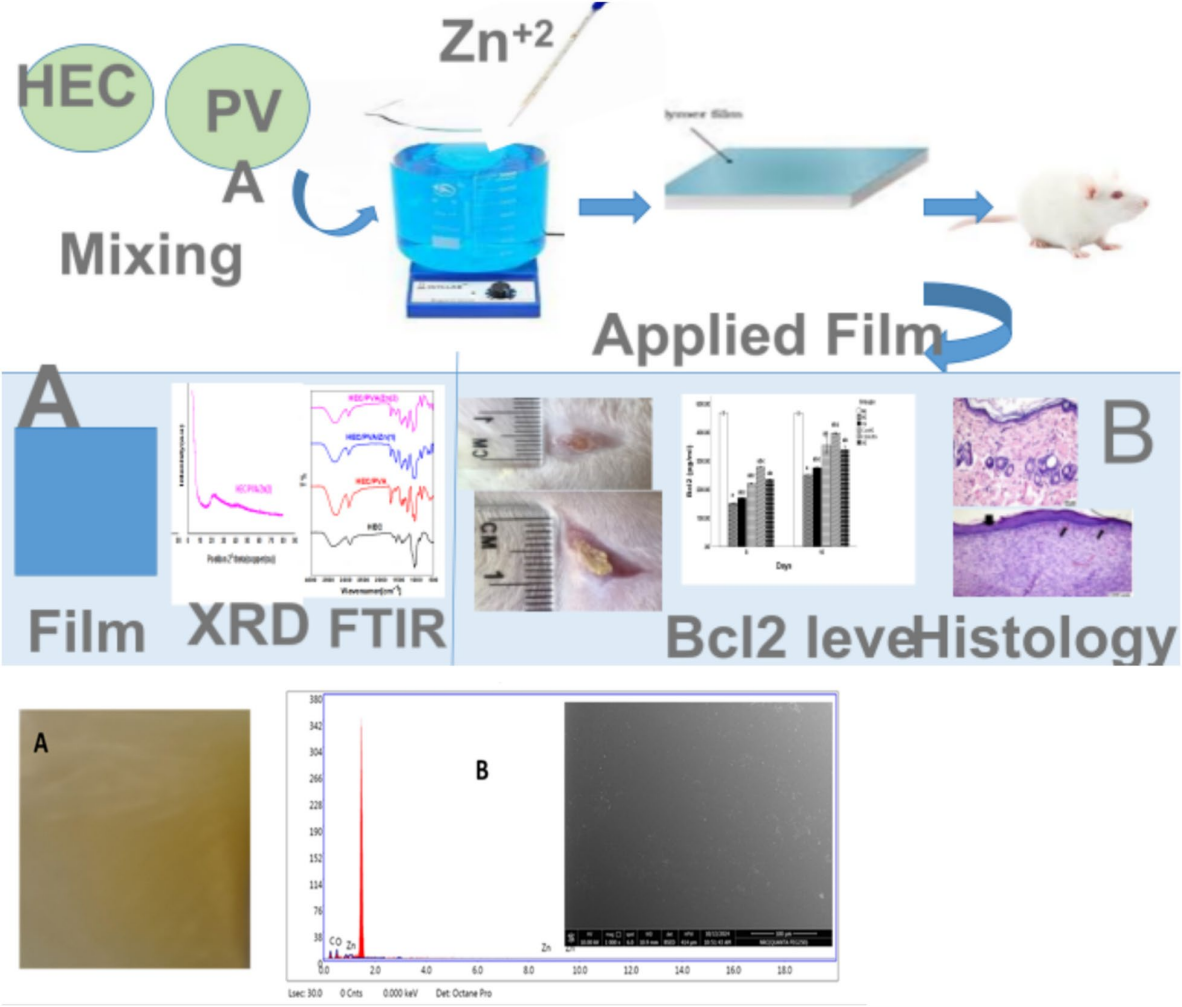
HEC/PVA.Zn film preparation.

The film has been prepared by casting method based on HEC/PVA in presence of different ratios of (2%) zinc acetate (1 and 1.5 ml). homogeneous and relatively smooth surface has obtained. HEC and PVA have adhesive properties due to presence of COOH and OH group that capable of hydrogen bond formation. As will Zn plays two roles in the prepared film; i) as a cross linker agent to gather HEC chains with PVA. ii) Acts as wound healing material due to its properties^30^. Figure 1. Shows HEC/PVA/Zn film preparation and represents the prepared film as a homogenous smooth surface that forced bySEM image and EDX with photo image of prepared film.

### FTIR analysis

**Fig. 2:**
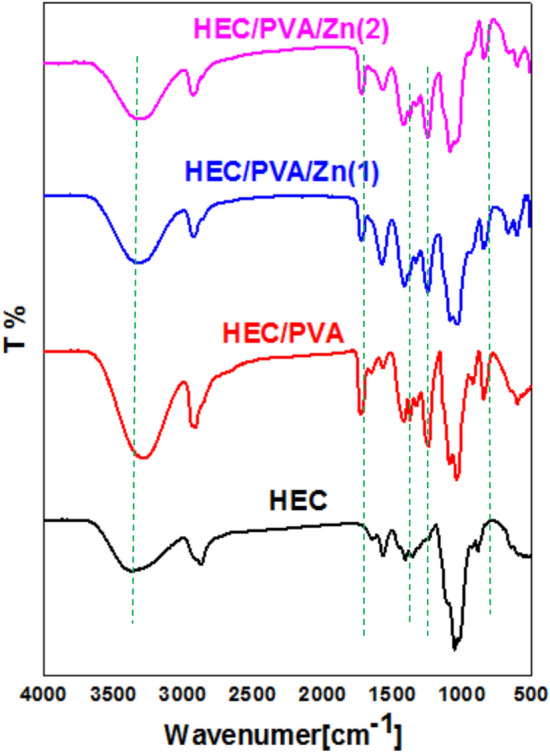
FTIR of HEC,HEC/PVA, HEC/PVA/Zn1 and HEC/PVA/Zn2.

FTIR analysis is a useful tool to study the chemical structural of different compounds. Figure 2 illustrates FTIR of HEC,HEC/PVA, HEC/PVA/Zn1(with 1 ml of 2% Zn solution) and HEC/PVA/Zn2 (with 1.5 ml of 2% Zn solution). HEC shows different peaks at 3500 cm^−1^, 2900 cm^−1^, 1650 cm^−1^ and at 1100 cm^−1^ corresponding to OH stretching, CH stretching, C=O absorbed water and ether linkage C–O–C. the mixing of HEC with PVA produced new peak at 1700 cm^−1^ and at 1200 cm^−1^ due to C=O and C–O. After Zn addition the characteristics peaks of HEC/PVA increased due to Zn cross-linker affect that increase HEC/PVA interaction^31^.

### X-ray diffraction

**Fig. 3:**
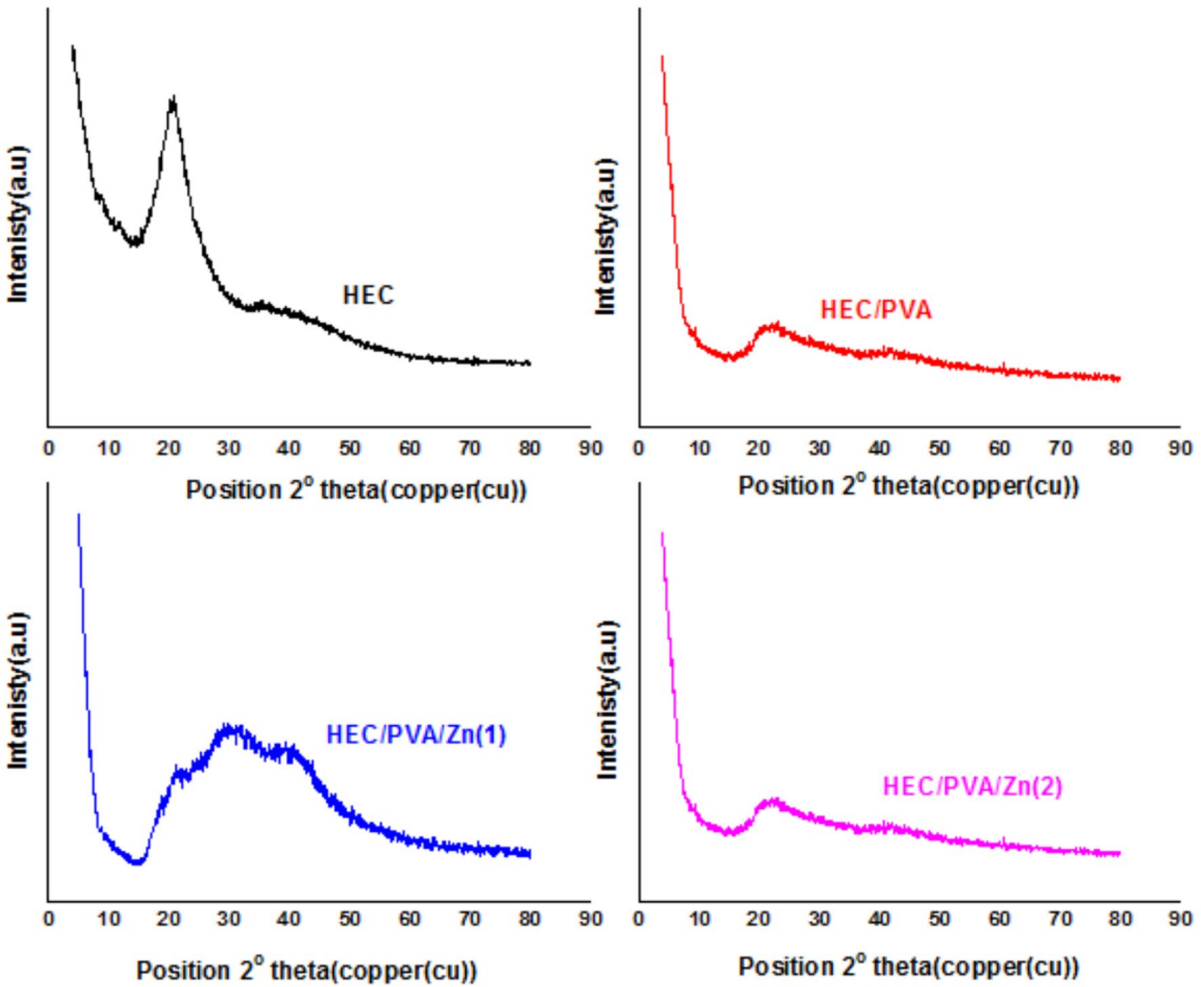
XRD of HEC,HEC/PVA, HEC/PVA/Zn1 and HEC/PVA/Zn2.

XRD pattern reflect the interaction effect between the different polymers. Figure 3 show XRD pattern of HEC, HEC/PVA, HEC/PVA/Zn1 and HEC/PVA/Zn2. HEC shows a distinctive peak at 2θ = 20°,attributed to crystalinity region. After PVA mixing this peak became slightly appear due to Zn particles distribution in the polymer chain^32^.

### Biochemical inspection

Wound healing is a natural response for tissue injury that treats tissue uniformity and necessitates the synthesis of a new extracellular matrix^33^**.** This process is frequently allocated into three phases: the inflammatory phase [hemostasis and inflammation], the proliferative phase [granulation, wound contraction, and epithelialization], and the regeneration phase [maturation and collagen tissue reconstruction]^34^. Topical zinc therapy has demonstrated promising outcomes in improving wound healing through accelerating biochemical and molecular processes in hemostasis, inflammation and cell proliferation^35^. Thus, applying topical zinc treatments for promoting wound healing enhances quality of life and offers therapeutic advantages.

The current semisolid preparations like creams and ointments have limitations, as they do not ensure persistent contact with the skin surface and can be easily wiped off by patient’s clothes^16^. Hence repeated application is required in case of chronic diseases^15^. Also these leave a sticky and greasy feel after application leading to poor patient compliance^36,37^. Therefore there is a need for development of a dosage form which permits less frequent dosing by maintaining a close contact with the skin for prolonged time period thereby improving the patient compliance. A Topical film-based formulation is a novel dosage form where drugs are incorporated into thin films that dissolve quickly in the mouth or on the skin, offering convenient and fast drug delivery^38^. In this study, we demonstrated the effectiveness of Zinc/cellulose (C-Zn) films in treating burn skin injury. C-Zn films greatly accelerated the healing of burn wounds through hindering inflammation, promoting collagen synthesis, inhibiting apoptosis, stimulating neogenesis, and remodeling.

**Fig. 4:**
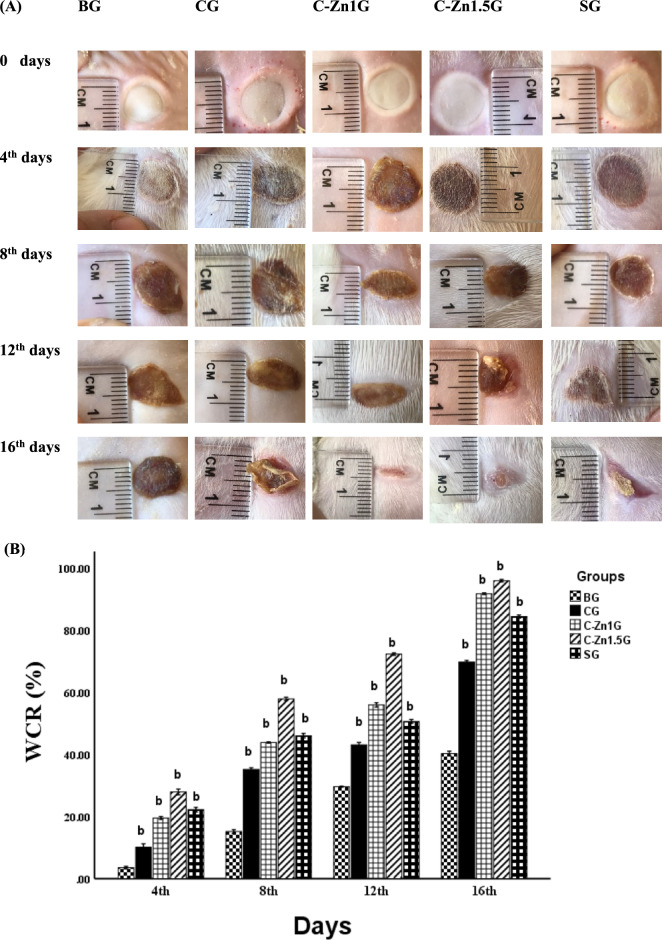
(**A**) Pictorial representation of rat burn skin injury at different times and treatments, (**B**) Comparative efficacy of different treatments on burn wound healing in various times n = 6. Data are denoted as mean ± SEM. b, P < 0.05 vs. BG.

In the current study, representative images of burn injuries in *Wistar* rats have been displayed in Fig. 4A. Wound contraction assay was carried out for evaluating faster burn wound healing. Time-dependent and treatment-dependent variations in the rate of wound contractions were identified throughout the 16-day observation for wound contraction rates (WCR) in all treated and untreated groups, as illustrated in Fig. 4B.

On various days of treatments, the wound contraction rates of the different groups were contrasted with the burn group. The burn wound contraction rates on days 8, 12, and 16 were significantly higher in all treated groups than in the burn group, possibly this could be attributed to an accelerated burn wound healing process in the treated groups, particularly in the C-Z1.5G group. The percentage of changes in wound areas at different times and treatments were illustrated in Table 3. These results implied that C-Zn1.5 films have a significant positive effect on the burn wound closure rate.

**Table 3:** The percentage of changes in wound areas at different times and treatments.

Groups	Time interval			
	4th days	8th days	12th days	16th days
CG	− 8.74%	− 23.56%	− 19.90%	− 49.30%
C-Zn1G	− 17.08%	− 33.75%	− 37.38%	− 86.03%
C-Zn1.5G	− 24.70%	− 50.28%	− 60.58%	− 93.10%
SG	− 20.24%	− 36.34%	− 29.75%	− 73.77%

Pro-inflammatory cytokines affect various processes at the injury site, involving immune response modulation, fibroblast chemotaxis, extracellular matrix proteins synthesis and degradation, promotion of keratinocyte and fibroblast proliferation^39^ **.**

**Fig. 5:**
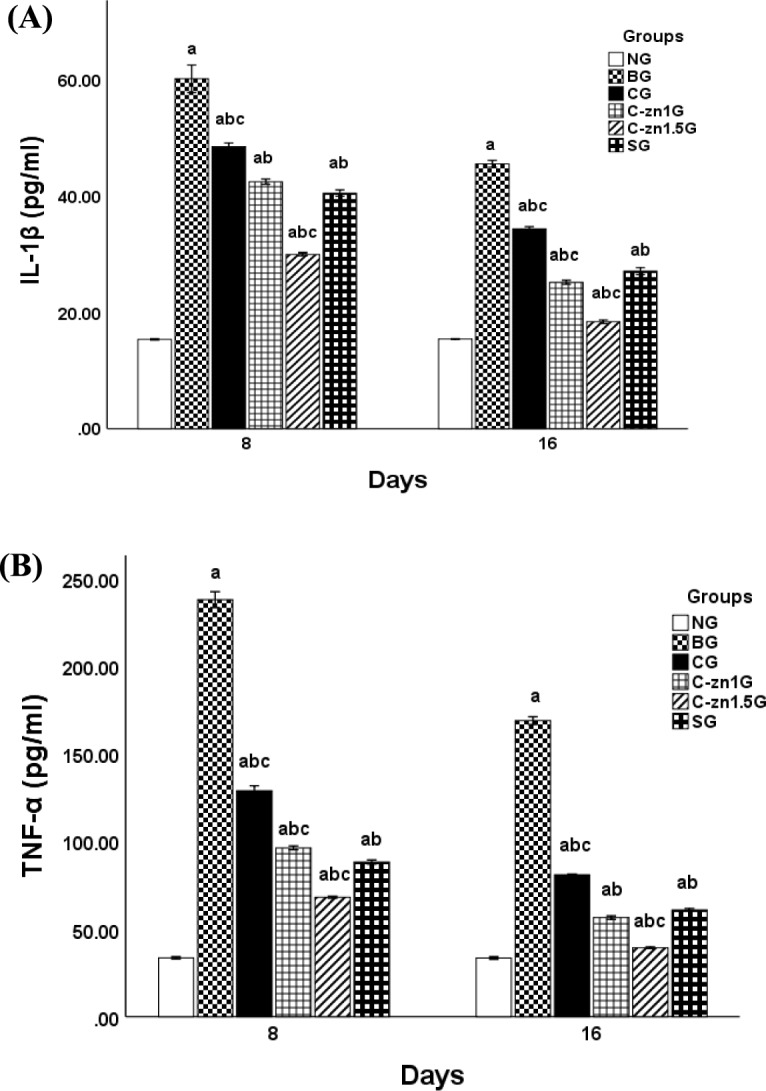
Effect of different topical treatments on skin inflammatory mediators levels in burnt rats (n = 6): (**A**) skin IL-1β level and (**B**) skin TNF-α level. Data were denoted as mean ± SEM. a, P < 0.05 vs. NG; b, P < 0.05 vs. BG; c, P < 0.05 vs. SG.

After 8-days post-burn injury, burnt rats revealed a notable increase in IL-1β and TNF-α levels in their skin tissues by (291.13%) and (609.74%) respectively (Fig. 5A and B) in contrast to the NG. Whereas, as compared to the BG, all treated groups considerably recovered changed inflammatory markers levels [for IL-1β being: 19.44% for CG, 29.46% for C-Zn1G, 50.09% for C-Zn1.5G, and 32.74% for SG and for TNF-α being: 45.83% for CG, 59.52% for C-Zn1G, 71.35% for C-Zn1.5G, and 62.91% for SG] (Fig. 5A and B). Additionally, compared to SG, applying C-Zn1.5 films significantly reduced skin IL-1β and TNF-α levels but C-Zn1G manifested the same potency as SG in decreasing skin IL-1β level (Fig. 5A and B).

While at 16-days, burnt skin tissues of BG illustrated a significant elevation of rats’s IL-1β and TNF-α level by (194.72%) and (404.9%) respectively when compared with the non-burnt group as denoted in Fig. 5A and B. However, topical application of different treatments significantly decrease levels of studied inflammatory markers contrasting to BG [IL-1β; 24.58% for CG, 44.57% for C-Zn1G, 59.5% for C-Zn1.5G, and 40.34% for SG] [TNF-α; 52.1% for CG, 66.5% for C-Zn1G, 76.7% for C-Zn1.5G, and 63.9% for SG] (Fig. 5A and B). Also, C-Zn1G and C-Zn1.5G significantly reduced IL-1β and TNF-α skin levels comparing to SG.

In agreement with our results, Tammam et al.^40^ detected that the induction of burn provoked markedly higher levels of TNF-α and IL-6 than those in the control group. Besides, Irfan et al.^41^ have documented that burnt skin tissues exhibit elevated expression levels of IL-1β and IL-6 mRNA. This could be attributed to burn injuries can trigger inflammatory responses^42^ and stimulate pro-inflammatory cytokines production such IL-1β and TNF-α that are crucial for wound reparation^43^ .

Conversely, diminished skin levels of pro-inflammatory cytokines in diverse treated groups according to our results indicated that the inflammation gradually reduced. Gaweł et al.^44^ reported Zinc acts as an antioxidant and anti-inflammatory that can attenuate wound inflammation through declining the mRNA expression of pro-inflammatory cytokines (IL-1β and TNF-α) via modulating Nuclear Factor-Kappa B (NF-κB), a transcription factor that controls oxidative stress and regulates pro-inflammatory responses^45^***.*** In addition, Silver sulfadiazine exhibited efficiency in treatment of burn injury through its anti-inflammatory effects^46^***.***

One essential constituent of the extracellular matrix is collagen1^47^**.** Collagens mediate several important processes in the wound environment, including granulation tissue development, angiogenesis, regulation of inflammation, platelet aggregation, and re-epithelialization in an integrin signaling dependent manner^48^**.**

**Fig. 6:**
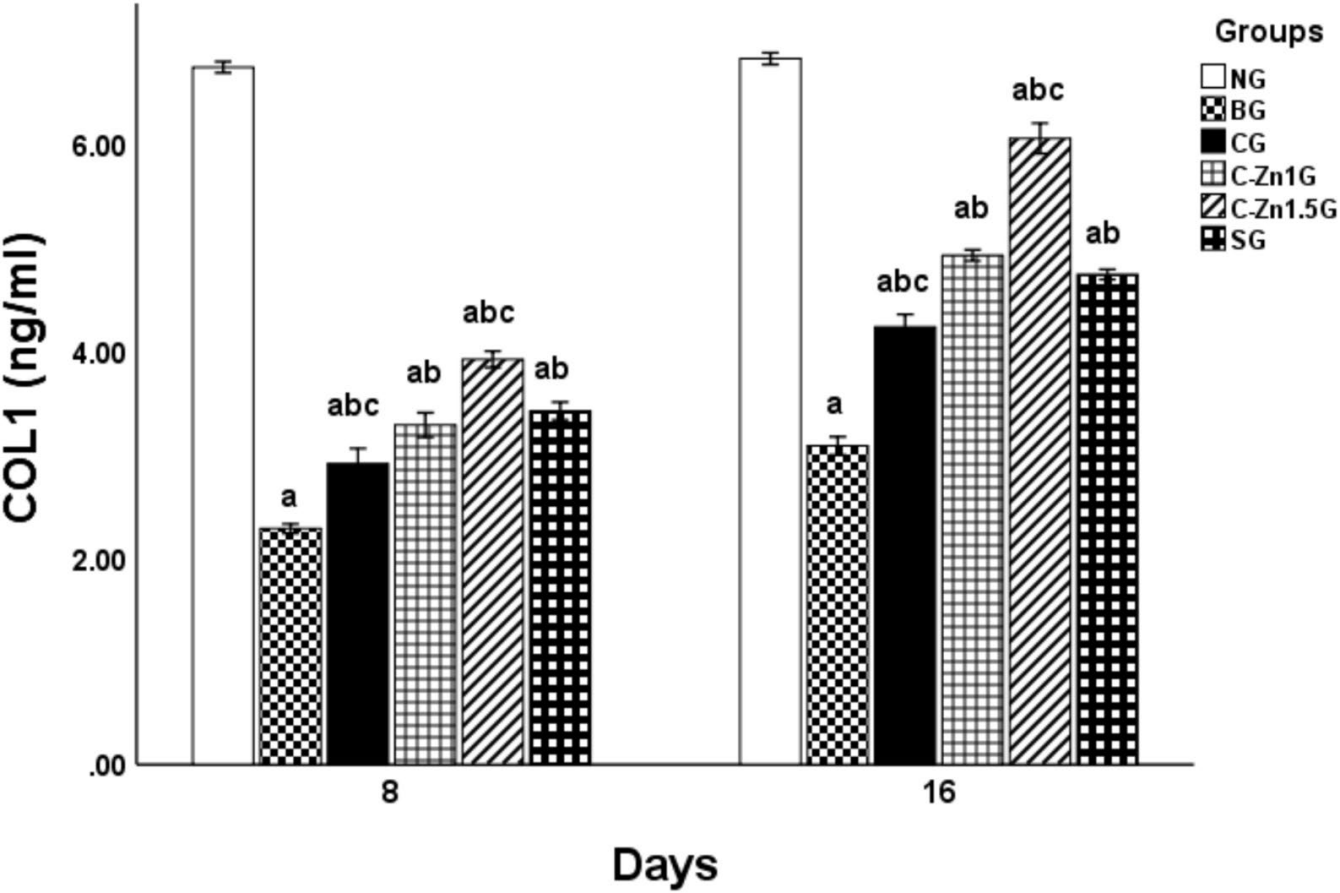
Effect of different topical treatments on skin collagen 1 level in burnt rats (n = 6). Data are denoted as mean ± SEM. a, P < 0.05 vs*.* NG; b, P < 0.05 vs. BG; c, P < 0.05 vs. SG.

In the present study, after 8-days post-thermal injury, BG illustrated a considerable decrease in collagen 1 skin level (66.16%) (Fig. 6) when compared with the NG. On the other hand, relative to BG, topical application of Silvazine cream and Zinc-cellulose films with varying concentrations markedly promoted the amount of collagen 1 in the skin [27.52% for CG, 43.92% for C-Zn1G, 71.74% for C-Zn1.5G, and 49.8% for SG] (Fig. 6). Additionally, treatment with C-Zn1.5 film considerably raised the level of skin collagen 1 in comparison to SG, whereas C-Zn1G manifested the same efficacy as SG in elevating this marker’s level (Fig. 6).

Even though, at 16-days after burn induction, Fig. 6 demonstrated that animals’ dorsal side burns resulted in a remarkable attenuation in the skin collagen 1 amount (54.9%) compared to the NG. However, in contrast to BG, usage of Silvazine cream and C-Zn films induced significant improvement of collagen 1level in skin tissues [37.31% for CG, 59.8% for C-Zn1G, 96.5% for C-Zn1.5G, and 53.8% for SG]. As well, utilization of C-Zn1.5 films considerably elevated skin collagen 1level but; C-Zn1G was just as successful as SG at boosting the level of this marker (Fig. 6).

Our findings suggested that reduction in collagen 1 amount in burnt groups may be related to the initial inflammatory reaction that is predominantly dominated by the pro-inflammatory macrophage M1 phenotype in response to burn wounds^49,50^. According to prior investigations, fibroblasts cocultured with M1 macrophages have diminished collagen synthesis and proliferation^51,52^**.** M1 macrophages release a variety of proteolytic enzymes to degrade extracellular matrix, which includes collagens, elastin, and fibronectin**.**

Contrarily, our results indicated that the topically applicate C-Zn1 and C-Zn1.5 films induced the level of collagen 1 protein in burnt skin. These results are in accordance with Li et al.^25^, who illustrated that the released Zn^2+^ from zinc-doped Prussian blue (ZnPB) upregulated gene expression of COL-I and COL-III in addition to downregulating the gene expression of IL-1β in NIH-3T3 cells that can stimulate collagen deposition, and hinder inflammatory mediators to encourage wound healing. Augmented fibroblast infiltration and improved epithelialization were also recognized in the zinc-treated rabbits^53^*.* Moreover, zinc is implicated in metalloproteinases activities that responsible for cleavage of propeptides of procollagen molecules^54,55^ a step that controls the rate of collagen synthesis^56^**.**

**Fig. 7:**
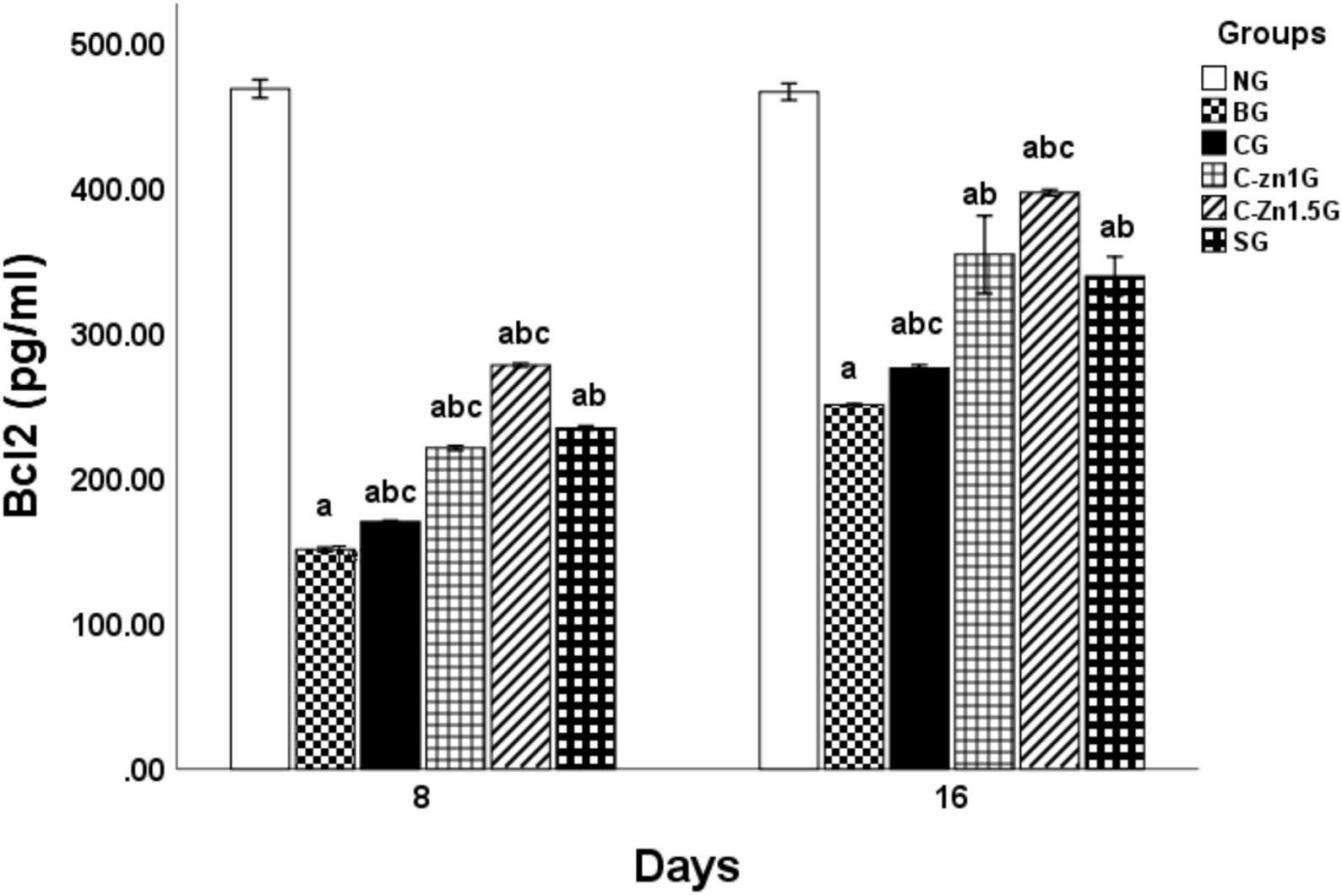
Effect of different topical treatments on skin Bcl2 level in burnt rats (n = 6). Data are denoted as mean ± SEM. a, P < 0.05 vs. NG; b, P < 0.05 vs. BG; c, P < 0.05 vs. SG.

As denoted in Fig. 7, after 8 days postoperative, induction of burn caused a significant lessening in rats’s skin Bcl2 level (67.73%) in comparison with the non-burnt group. However, topical utilization of C, C-Zn1, C-Zn1.5 films and Silvazine cream substantially resolved burn injury-prompted apoptosis as manifested via an increase in Bcl2 level contrasting with BG [12.68% for CG, 46.26% for C-Zn1G, 84% for C-Zn1.5G, and 55.29% for SG] (Fig. 7). Equating with SG, C-Zn1.5G illustrated an enormous rise in Bcl2 level in burnt skin.

Meanwhile, at 16 days post burn induction, a marked reduction in skin Bcl2 level (46.22%) was recorded in the group with thermal injury as compared with NG (Fig. 7). Nevertheless, C, C-Zn1G, C-Zn1.5G, and SG, all displayed a considerable rise in Bcl2 in comparison with BG [15.87% for CG, 41.28% for C-Zn1G, 58.28% for C-Zn1.5G, and 35.32% for SG] (Fig. 7). Comparing with SG, C-Zn1.5G illustrated a significant increase in Bcl2 level of burnt skin whereas C-Zn1G is just as effective as SG at raising Bcl2 levels (Fig. 7).

These outcomes are consistent with former study by Tammam et al.^40^, which clarified that in burnt skin, the level of anti-apoptotic marker Bcl2 decreased while, the levels of the pro-apoptotic indicators Bax and caspase-3 increased. Burn-induced oxidative stress rose the generation of oxygen free radicals, which were essential in triggering cell apoptosis^57,58^ by activating the P53 protein. P53 protein triggered apoptosis via down-regulating Bcl2 level and activating the Bax^59,60^. However, by raising the amount of Bcl2, topical using of C-Zn films significantly counteracted the apoptosis caused by burn damage in skin tissue. Lin et al.^61^ found that Zn treatment inhibits apoptosis in mice with spinal cord injuries, via upregulating Bcl-2 level. Zinc’s anti-apoptotic action is likely due to its cytoprotective properties against oxidative stress and bacterial toxins, which may be mediated by the antioxidant activity of metallothioneins that are rich in cysteines^22^**.**

### Molecular investigations

Vascular endothelial growth factor (VEGF) and transforming growth factor β (TGF-β) are angiogenic cytokines that aid in the growth of blood vessels and the multiplication of endothelial cells; they are involved in wound angiogenesis^62,63^, and VEGF also controls integrin receptors for the formation of new blood vessels^64^. Besides TGF-β, it promotes the proliferation of endothelial cells and the deposition of extracellular matrix by serving as a chemoattractant for fibroblasts, neutrophils, and macrophages at the wound site^65^. The current study demonstrated that the expression of angiogenesis-related genes, including both VEGF and TGF-β, shared the same expression pattern in rat skin tissues in different studied groups.

**Fig. 8:**
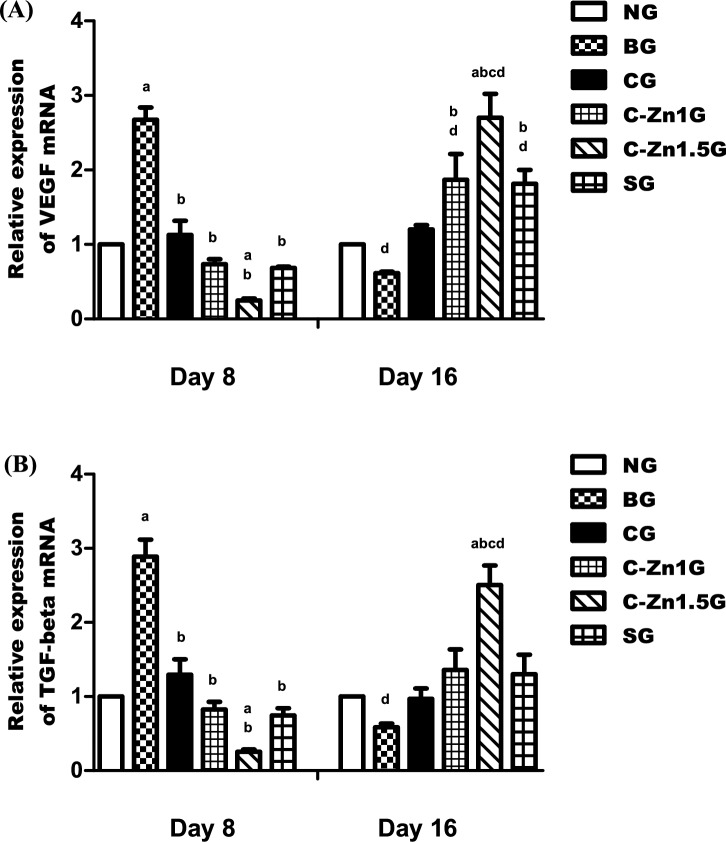
(**A**,**B**) Real-time polymerase chain reaction for angiogenesis-related genes VEGF and TGF-β on day 8 and 16 of the experiment. Data were expressed as Mean ± SEM. a, p < 0.05 vs. NG; b, p < 0.05 vs. BG; c, p < 0.05 vs. SG; d, p < 0.05, day 8 vs. day 16.

At day 8, both VEGF and TGF-β genes were significantly increased (p < 0.05) in the BG by about 160% and 168% than that of the NG (Fig. 8A,B). On the other hand, VEGF and TGF-β genes were significantly decreased in all treated groups, especially the C-Zn1.5G (by about 90% and 91%), where the expression level was significantly lower than the corresponding CG (by about 57% and 55%), C-Zn1G (by about 72% and 71%), and SG (by about 73% and 74%) compared to BG (p < 0.05) (Fig. 8A,B). The C-Zn1.5G and C-Zn1G showed non-significant impacts in both the VEGF and TGF-β genes when compared to the SG (Fig. 8A,B).

At day 16, VEGF and TGF-β genes (by about 39% and 42%) were decreased in the BG compared with the NG (Fig. 8A,B). While C-Zn1.5G (by approximately 340% and 313%) significantly elevated expression levels of both genes (VEGF and TGF-β), C-Zn1G (by approximately 203%) and SG (by approximately 195%) showed significant increases in the VEGF gene only in comparison with the BG (p < 0.05) (Fig. 8A,B). The C-Zn1.5G showed significantly higher levels of both VEGF and TGF-β gene expression (p < 0.05) than the SG, and the C-Zn1G showed the same effect as the SG (Fig. 8A,B).

Our findings concur with those of Yan et al.^66^, who proposed that reduced expression levels of VEGF and TGF-β due to tissue hypoxia in burns is the primary cause, which results in no new blood vessel formation and decreased epithelization, and de Araújo et al.^18^, who demonstrated that on day 14 of the experimental model, the animals treated with the chitosan-zinc complex had higher mRNA expression for TGF-β and VEGF. Furthermore, Hussein et al.^67^ reported that normal wound rats (GIII) treated with CS/ZnO membranes showed a considerable increase in TGF-β and VEGF gene expressions in comparison to the normal non-treated group (GI). Another study found that bisacurone gel significantly raised VEGF levels in comparison to control, which suggested endothelial cell migration, vascular permeability, and the development of new blood vessels at the site of damage. Additionally, bisacurone gel raised TGF-β expressions, suggesting that it stimulates fibroblast migration and proliferation and has a role in controlling the production of collagen during wound healing . These results demonstrated that C-Zn1.5 treatment significantly increased angiogenesis, endothelial cell migration, and proliferation by up-regulating VEGF and TGF-β expression, suggesting ameliorated burn wounds.

Matrix metallopeptidases (MMPs) are essential for maintaining the equilibrium between the synthesis and degradation of extracellular matrix (ECM) in the latter phases of wound closure. Also, tissue inhibitors of metallopeptidase (TIMPs) and their naturally occurring inhibitors decrease the action of MMPs. Consequently, they cooperate to provide appropriate tissue remodeling in the burn wound^68^**.** A major contributor to ECM turnover, TIMP2 mediates the activity of MMPs, especially MMP2^69^**.** Additionally, MMP2 probably contributes significantly to the latter phases of wound repair by reducing inflammation, which is an important step due to chronic inflammation inhibits healing if it persists for a number of days^70^**.** MMP2 reduces inflammation by deactivating and truncating MCP-3, a CC chemokine that stimulates leukocyte chemotaxis. This prevents an *in vivo* inflammatory response from initiating and completely eliminates any pre-existing inflammation^71^**.**

**Fig. 9:**
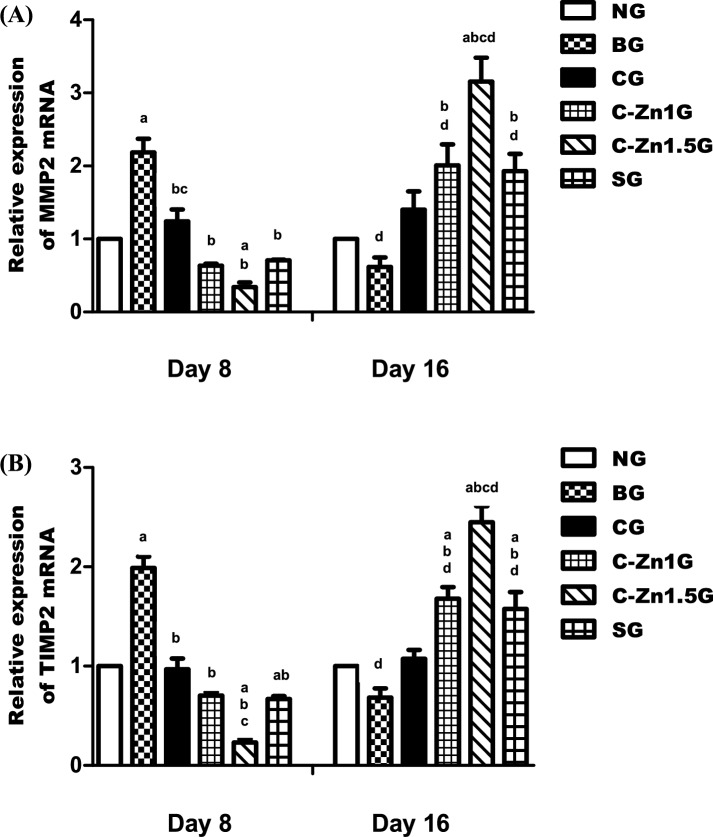
(**A**,**B**) Real-time polymerase chain reaction for matrix remodeling related genes MMP2, and TIMP2 on day 8 and 16 of the experiment. Data were expressed as Mean ± SEM. a, p < 0.05 vs. NG; b, p < 0.05 vs. BG; c, p < 0.05 vs. SG; d, p < 0.05, day 8 vs. day 16.

At day 8, matrix remodeling related genes including, MMP2 and TIMP2, changed over time synchronously. The present study revealed their expression all significantly increased in the BG by about 126% and 98% more than the NG (p < 0.05) (Fig. 9A,B). On the other side, significantly decreased levels of MMP2 and TIMP2 expression were observed in all the groups (CG (by around 45% and 51%), C-Zn1G (by around 72% and 65%), and SG (by around 73% and 67%)), especially in the C-Zn1.5G (by around 85% and 88%), lower than those found in the BG (p < 0.05) (Fig. 9A,B). When compared to the SG, the C-Zn1.5G exhibited a significant decline in TIMP2 expression levels (p < 0.05), while the C-Zn1G had non-significant effects in both the MMP2 and TIMP2 genes (Fig. 9A,B).

At day 16, in this study, the expression levels of MMP2 and TIMP2 genes declined (about 39% and 32%) in the BG compared with the NG (Fig. 9A,B). Nonetheless, the expression levels of MMP2 and TIMP2 genes in the C-Zn1.5G (roughly 413% and 259%) showed a significant increase compared to the C-Zn1G (roughly 227% and 146%) and SG (roughly 215% and 132%) in comparison with the BG (p < 0.05) (Fig. 9A,B). In comparison to the SG, the C-Zn1.5G exhibited significantly greater levels of MMP2 and TIMP2 gene expression (p < 0.05), whereas the C-Zn1G exhibited the same potency as the SG (Fig. 9A,B).

According to Pilar et al.^72^, MMP2 and MMP9 have a role in cell migration and re-epithelization, which is consistent with our finding. However, MMP2 and MMP9 activities appear to be higher in non-healing wounds than in healing wounds, and blocking these enzymes enhances the healing process^73^. Zhou et al.^74^ stated that the balance between MMPs and TIMPs primarily regulates the synthesis and degradation of ECM, which undermines the stability of the ECM and encourages the development of skin damage. Previous studies have shown that zinc is an essential cofactor for several MMPs involved in the wound healing process^75^. Zinc can inhibit the activity of MMPs by directly interacting with them. First, excessive zinc binding changes the structure of MMP proteins or forms a bridge between zinc and hydroxide that stops the catalytic site of MMPs from working. However, the concentration of zinc may affect its activity. Second, the enzyme may become inactive due to metal interaction, which can change protein consolidation at the catalytic region. Additionally, it has been shown that zinc accumulation increases oxidative stress in ischemic microvessels by first making superoxide anion stronger, which then increases the activities of MMP9 and MMP2^76^**.** According to these results, C-Zn1.5 treatment was effective because it was associated with accelerated burn wound healing by upregulating the expression of remodeling-related genes MMP2 and TIMP2.

### Histopathological investigation

**Fig. 10:**
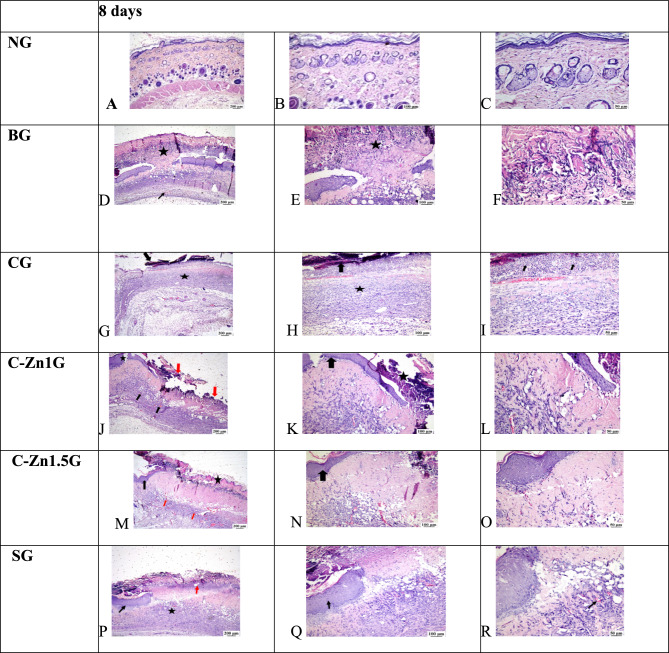
At 8 days: (**A**–**C**) Photomicrograph of NG showed normal histological structure of epidermis and dermis layers; (**D**–**F**) Photomicrograph of BG displayed severe necrosis with intense inflammation and edema (star (**D**,**E**)) with heavy inflammatory cells infiltration and intense neutrophils (star (**E**)), congested blood vessels in the deep dermal layer (arrow (**D**)); (**G**–**I**) Photomicrograph of CG viewing sero-cellular crust with absence of epithelial cover with filling of the wound gap (arrow (**G**–**I**)) with inflamed granulation tissue (star (**G**,**H**)), (**I**) showed intense neutrophils infiltration (arrow) beneath the sero-cellular crust with inflamed granulation tissue filling of the wound gap; (**J**–**L**) Photomicrograph of C-Zn1G illustrated sero-cellular crust (red arrow (**J**), star (**K**)) with partial re-epithelization at the wound edge (star (**J**) and arrow (**K**)) and less inflamed granulation tissue filling the wound gap with numerous blood vessels (black arrow (**J**)). (**M**–**O**) photomicrographs of C-Zn1.5G presenting sero-cellular crust (star (**M**)) with partial re-epithelization with keratin at the wound edge (black arrow (**M**,**N**)) and well-developed collagen rich granulation tissue filling the wound gap with numerous blood vessels (red arrow (**M**)), (**P**–**R**) photomicrograph of SG screening sero-cellular crust (red arrow (**P**)) with partial re-epithelization at the wound edge (black arrow (**P**,**Q**)) with collagen rich less inflamed granulation tissue filling the wound gap (star (**P**)), newly formed blood capillaries were noticed ( black arrow (**R**)).

At 8 postoperative days the microscopic examination of skin tissue section from the NG showed normal skin histological structures (Fig. 10A–C). In contrast, the injured skin area of the BG revealed thick sero-cellular crust that composed of necrotic tissue debris and inflammatory exudates covering the surface of the wound with intense inflammatory cells infiltration in addition to severely congested blood vessels were recognized in the deep dermal layer (Fig. 10D–F). Poor burn healing was noticed in the CG, displaying a complete loss of epithelial cover with intense neutrophils infiltration beneath the sero-cellular crust. The burnt wound site filled with inflamed granulation tissue with poor vascularization (Fig. 10G–I). Zinc-cellulose-1 group seemed similar to SG but with more inflammatory reaction (Fig. 10J–L). Zinc-cellulose 1.5group revealed a sero-cellular crust with partial re-epithelization with keratin at the burn wound edge and the underlying gap was filled with well-developed collagen rich granulation tissue which enclosed frequent capillaries and less inflammation (Fig. 10M–O). The Silvazine group illustrated moderate enhancement in healing process as the examined sections revealed partial re-epithelization with keratinization at the injury edge beneath the crust with collagen rich granulation tissue filling the wound gap. Numerous recently formed blood capillaries were remarked (Fig. 10P–R).

**Fig. 11:**
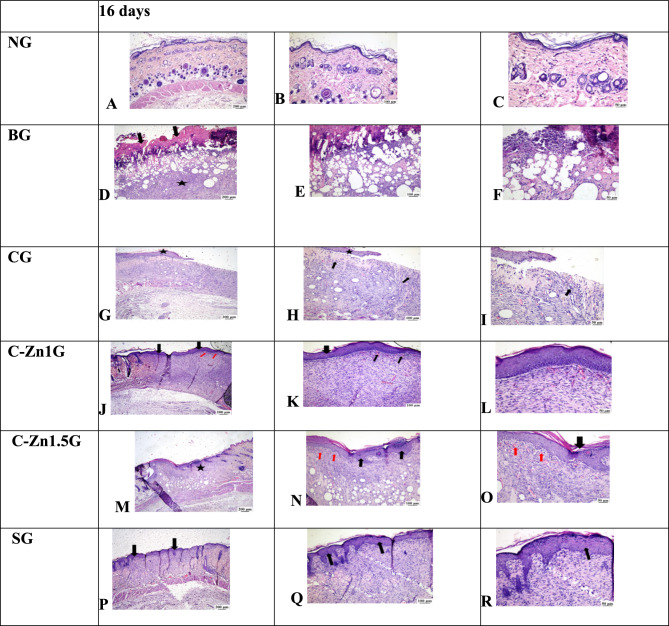
At 16 days: (**A**–**C**) Photomicrograph of skin with normal histological structure of epidermis and dermis layers in normal control group; (**D**–**F**) Photomicrograph of BG skin showing thick sero-cellular crust and absence of re-epithelization (arrow (**D**)) with inflamed granulation tissue in the wound area (star (**D**)); (**G**–**I**) Photomicrograph of blank cellulose group showing partial re-epithelization (star (**G**,**K**) and arrow (**I**)) with presence of inflamed and well vascularized granulation tissue in the wound area, note inflammatory cells (arrow (**K**,**I**); (**J**–**L**) Photomicrograph of cellulose-Zn1 group showing complete re-epithelization (black arrow (**J**) and thick arrow (**K**)) with less inflamed organized tissue filling the wound gap, note the presence of numerous capillaries under the newly formed epithelium (red arrow (**J**) and thin arrow (**K**)); (**M**–**O**) Photomicrograph cellulose-Zn1.5 group showing marked wound closure with complete re-epithelization (star (**M**) and black arrow (**N**,**O**)) and filling of the wound gap with well vascularized collagen rich organized tissue, note blood capillaries under newly formed epithelium (red arrow (**N**,**O**)); (**P**–**R**) Photomicrograph of Silvazine group showing complete re-epithelization (arrow (**P**–**R**)) with collagen rich organized tissue filling the wound gap.

However, at 16 days post thermal injury, the microscopic examination of skin tissue section from the NG showed normal skin histological structures (Fig. 11A–C). Conversely, BG manifested evidence of delayed healing as absence of re-epithelization with thick sero-cellular crust and haphazardly arranged granulation tissue with intense inflammatory reaction filling the wound site (Fig. 11D–F). The blank cellulose group demonstrated a mild degree of enhancement, exhibiting partial re-epithelization at wound edge with moderate inflammatory granulation tissue in the injury area (Fig. 11G–I). Zinc-cellulose 1 group displayed also complete re-epithelization with less inflamed organized tissue filling the wound gap. Many blood capillaries under the newly formed epithelium were recognized (Fig. 11J–L). Marked wound remodeling and closure with complete re-epithelization with keratinization were observed in C-Zn1.5G, the wound gap was filled with well vascularized collagen rich organized tissue with mild inflammatory response (Fig. 11M–O). The Silvazine group exhibited marked improvement, the wound surface was covered with newly formed epithelium and the wound gap was filled with collagen rich and well vascularized organized tissue (Fig. 11P–R).

Long-term biocompatibility concerns for the synthesized formulation as burn wound healing agent including the need for extensive testing to ensure no adverse immune response or long-term toxicity, and the importance of evaluating its biodegradability and integration with host tissue over time. However, future perspectives include optimizing the formulation for different wound types, developing more advanced models for testing, and integrating it into innovative therapies like 3D bio-printing or bio-scaffolds for enhanced, scar-free healing.

This study had certain experimental limitations including anti-bacterial impact of C-Zn films needs to be detected. Different treatments under investigation need to be applicate on in vitro model to support the in vivo experiment. It is necessary to estimate more biomarkers to illustrate the positive impact of C-Zn films on burn wound healing as growth factors including epidermal growth factor and fibroblast growth factors. Also, additional investigations will be required using C-Zn in different forms as hydrogels and comparing their effects on burn healing with C-Zn films which will open new avenues for therapeutic interventions. Future researches need to focus on more comprehensive multidisciplinary approaches that address entire recovery process.

## Conclusion

In summary, treatment with C-Zn 1.5 film improved healing of burnt rats by suppressing pro-inflammatory cytokines levels IL-1β (*p<0.001)* and TNF-α (*p<0.001*), enhancing collagen synthesis (collagen 1 *p<0.001*), inducing the anti-apoptotic marker (Bcl2 *p<0.001*), and promoting re-epithelization with keratinization at 8 and 16 post-operative days. Also, on day 16, application of C-Zn 1.5 film increased the expression levels of the angiogenic genes VEGF and TGF-β ( ~ 340% and 313%, respectively), as well as the remodeling-associated genes MMP2 and TIMP2 (~ 413% and 259%, respectively). Our findings provided fully support for the beneficial use of C-Zn 1.5 film in the management of burn wounds by reducing inflammation, stimulating re-epithelization with keratinization, promoting collagen synthesis, triggering angiogenesis, and, ultimately, encouraging appropriate tissue remodeling.

## Data Availability

All data generated or analyzed during this study are included in this published article.
